# Quality of life of young clinical doctors in public hospitals in China’s developed cities as measured by the Nottingham Health Profile (NHP)

**DOI:** 10.1186/s12939-015-0199-2

**Published:** 2015-09-24

**Authors:** Ying Liang, Hanwei Wang, Xiaojun Tao

**Affiliations:** Department of Social Work and Social Policy, School of Social and Behavioral Sciences, Nanjing University, Nanjing, People’s Republic of China; School of Fine Arts, Nanjing Normal University, Nanjing, People’s Republic of China; College of Cultural Industries of Nanjing Art Institute, Nanjing, People’s Republic of China

**Keywords:** Nottingham health profile, Quality of life, Young clinical doctors, East china, Medical system

## Abstract

**Background:**

In contemporary Chinese society, obstacles such as frequent violence against medical workers and tense doctor–patient relationships affect the health of Chinese doctors. This study attempted to explore the quality of life (QOL) of young clinical doctors in public hospitals in China’s developed cities to study the psychometric properties of QOL and related risk factors of doctors’ health.

**Methods:**

This study sampled young doctors aged 15–45 in 18 public hospitals of three cities in East China (Shanghai, Nanjing, and Hangzhou, *N* = 762). The Nottingham Health Profile was used to measure QOL, the dependent variable of this study. Methodologies such as reliability analysis, mean comparison, and exploratory factor analysis were used to study related psychometric properties.

**Results:**

Almost 90 % of young Chinese clinical doctors have a bachelor’s degree or above. Approximately 70.4 % of the doctors have relatively low job titles. Among the sample, 76.1 % have a monthly income ranging from USD 326 to USD 1139, and 91.3 % work over eight hours daily. These respondents have poor sleeping habits and mental functions, but have relatively good physical functions. Being female, low education, low job title, low salary, and long work hours are factors associated with doctors’ poor QOL. Regression analysis results emphasize the great effect of high education on the improvement of QOL.

**Conclusions:**

Young clinical doctors in public hospitals in Chinese developed cities have poor QOL. Reforms on the current medical health system, improving the working environment of doctors and relieve their occupational stress should be required.

## Introduction

Medical work involves human services; as such, medical workers are at a high risk of occupational health hazards [1]. Physicians usually suffer from burnout [2], which is a long-term condition [3]. Other mental problems, such as emotional exhaustion, suicidal thoughts, depression, and anxiety, are also common among doctors [4–7]. For instance, Norwegian doctors express lower life satisfaction than the general population [8]. These psychological issues of medical professionals should be considered [9].

Doctors experiencing mental health problems may suffer from negative consequences, such as diminished productivity [10, 11], chemical abuse [1], excessive drinking [12], and low health and life satisfaction [13]. Young clinical doctors constitute the main growing force in hospital operations. These doctors undertake specific and intensive work but lack adequate clinical experience; as such, these doctors must satisfy numerous requirements in continuing studies. In addition, several complex factors contribute to the development of mental health problems among doctors. These factors can be divided into two aspects: internal individual factors and external contextual factors [14].

Internal individual factors include personal characteristics, psychological diathesis[15], sense of achievement, and occupational honor [16]. Individual devotion, attitudes, and practices toward the profession are also considered as the health predictors of health workers [17, 18]. By contrast, external contextual factors contributing to mental health conditions include working environments and occupational characteristics. Young doctors and medical college students consider learning burnout as an essential obsession [19]. The burnout symptoms experienced by doctors in different departments are associated with different features of their working environments [20]. The prevalence of burnout among resident doctors has been attributed to heavy workload and emotional distress [21]. Therefore, the mental health status of doctors is partly associated with the high demands of their job [3, 22]. Moreover, the regular contact between clinical doctors and their patients can cause tension, which further affects the mental condition of doctors. The doctor–patient relationship is a crucial factor affecting the work of doctors [23]. The unreasonable demand and complaint from patients can exacerbate the depression and suicidal tendency of doctors [24].

In a macro view, external contextual factors are parts of the medical care systems in China, where doctors work. China is currently in the stage of medical system transformation, as indicated by the increasing number of doctors and required improvements in service quality [25, 26]. Large Chinese patient populations dictate high medical demands. Limited available resources and government investments indicate that the medical service supply is indeed restricted. This situation shows that demands exceed supplies; as a consequence, these demands remain unsatisfied. Although the recent reform facilitated the expansion of insurance coverage and increased the proportion of reimbursement, the difficulties and expenses involved in visiting a doctor remain unresolved [27]. High out-of-pocket payments, cost escalation, and slow progress in the provision of adequate health insurance for all are considered as important challenges in the medical system reformation in contemporary China [28]. The complex medical system leads to the deterioration of the working environment of doctors.

In the context of this complex medical care system, the mental health of Chinese doctors is a major concern. Chinese doctors are dissatisfied with their professional lives [29, 30]. These doctors experience symptoms of depression [31], occupational stress [32], and anxiety [33]. Burnout, high emotional exhaustion, cynicism, and low perceived professional efficacy are strongly related to occupational pressure [34]. Therefore, the exacerbating mental health status of Chinese doctors requires further research [35].

Doctors’ mental disorders, such as anxiety, depression, burnout, and exhaustion, have been extensively investigated. Doctors’ negative moods caused by psychological illness have also been described, but their general health or quality of life (QOL) have been rarely explored on the basis of positive psychology [36]. Although studies on mental illnesses can provide considerable insights into these conditions, we cannot neglect the potential performance of mentally healthy doctors. A complete psychological health or health model should be free of psychopathological factors or physical diseases; this model should also include a progressive general well-being [37]. Thus, this study aims to develop new perspectives to evaluate the general health condition of doctors.

According to the World Health Organization (WHO), health not only indicates the absence of disease but also includes physical, mental, and social well-being [38]. And QOL is an important clinical health assessment tool. QOL is determined by the previous experiences, mental conditions, personalities, and expectations of subjects [39]. Although the standard definition has not been determined, QOL is the evaluation of the living environment and the satisfaction of individuals in their environment. QOL combines the expressed satisfaction and objective descriptions [40]. QOL is also a multidimensional concept affected by all life aspects of individuals. QOL measures the feeling or evaluates the individuals’ general life activities, including diseases, work, and social life [41]. QOL measurement introduces the humanistic element to health care. In addition, the information retrieved from QOL measurement results is highly useful to improve medical services [42]. The self-rated evaluation of respondents is sensitive to health changes; as such, the self-rated evaluation is considered as an efficient supplement in traditional, physiological, or biological measures; this tool is also helpful in the self-management of health [43]. Furthermore, QOL measurement results can be used in various applications [44], such as clinical experiments, clinical practices, decision making, and health management [45, 46]. Routine measurement can also effectively promote communication between doctors and patients; thus, the well-being of patients can be improved [47]. Therefore, health should be investigated on the basis of QOL to provide valuable insights into this condition observed among doctors.

 Scholars used an open-access database to compare the health-related QOL of Chinese doctors, nurses, and residents of the same age. It is found that the scores of doctors and nurses are less than those of residents [48]. However, these scholars have considered doctors and nurses in a homogeneous group and have not yet to investigate socio-economic determinants. The current study aims to fill the research gaps by sampling public hospitals in three relatively developed cities in East China. In this study, the following questions are addressed: What are the social, economic, and working conditions of the young clinical doctors working in the public hospitals in the developed cities in China? What is the status of the QOL of these doctors? What are the related psychometric properties? What are the factors affecting their QOL? This study also aims to (1) provide large-scale empirical data on the QOL of the young clinical Chinese doctors working in public hospitals; (2) investigate the socio-economic determinants of the QOL of doctors and the features of the QOL of this professional group; and (3) propose beneficial measures to health managers and related medical care institutions to improve the QOL of the doctors by analyzing the potential causes of the health conditions of these young clinical Chinese doctors.

## Methods

### Sampling process

The data used in this study were from the public hospitals of the relatively developed cities in East China. The investigation was conducted in March and April 2014. First, the purposive sampling method yielded 3 out of the 18 cities in the Yangtze River Delta (Shanghai, Nanjing, and Hangzhou). The Yangtze River Delta is a fan-shaped alluvial plain located in the estuary of the Yangtze River. Shanghai is the leading city, and the area consists of 18 cities in Zhejiang and Jiangsu provinces. This region is a rapidly developing area in China. According to the 2014 statistical yearbook issued by the National Bureau of Statistics of China (NBSC), the gross domestic product (GDP) of this region in 2013 ranked the highest, and the per capita GDP has exceeded USD 10,000. The living standard in this area is higher than that in other regions [49]. Therefore, the three cities can be considered as the representative of the developed cities in China to provide insights into the health status of the young clinical doctors working in these developed cities.

Second, when selecting the hospitals, we adopted a multi-stage random sampling method. Considering that the hospitals in each city are divided into three different levels, namely, provincial, municipal, and county, and that available resources, such as time and funds, are limited, we decided to select two hospitals in different levels in each city. As a result, six hospitals, including two provincial, two municipal, and two county hospitals, were randomly selected in each city. Eighteen hospitals were included in the samples.

Third, in accordance with the confidentiality principle in social science investigation, our questionnaires were anonymously filled out. In each hospital, 50 respondents were randomly selected from the general population. The inclusion criteria were as follows: clinical doctor, aged 15–45 years, and working in a public hospital. After the doctors expressed their consent to participate in the study, the trained investigators initiated the investigation. Each respondent independently filled out the questionnaire.

A total of 900 questionnaires were distributed; in each city, 300 forms were provided. The effective response rate was 84.67 % (of the total number of responses, 762 were considered valid). To determine whether the questionnaire is valid, the investigators examined whether all of the items were accurately, completely, and consistently answered before the data were encoded. If many items were missed or filled in error, the questionnaire was regarded as invalid and was thus discarded.

### Questionnaire

#### Basic information

Basic information contains the following variables: age, gender, education, marital status, job title 1, monthly income, and work hours per day. The specific categories under each variable are shown in Table 1.

**Table 1:** basic information of Chinese young clinical doctors

Categories	Frequency (N)	Percentage (%)
sex		
Male	445	58.4
Female	317	41.6
Education		
Junior college or below	77	10.1
Undergraduate	207	27.2
Graduate	253	33.2
Doctor/post doctor	225	29.5
Job titles		
Resident doctor	242	31.8
Physician	294	38.6
Associate chief physician	164	21.5
Chief physician	62	8.1
Marital status		
Unmarried	258	33.9
Married	395	51.8
Divorced or others	109	14.3
Monthly income		
< CNY 2000 (≈ USD 325)	101	13.3
CNY 2000–3000 (USD 326–488)	180	23.6
CNY 3000–5000 (USD 489–813)	231	30.3
CNY 5000–7000 (USD 814–1139)	169	22.2
>CNY 7000 (≈ USD 1140)	81	10.6
Work hours per day		
≤8 h	66	8.7
9–11 h	170	22.3
12–14 h	323	42.4
≥15 h	203	26.6

#### Nottingham Health Profile (NHP)

The NHP originated from the research instrument developed by the Department of Community Health at Nottingham University in 1975. NHP emphasizes the effect of ill-health on QOL [50]. The NHP is widely used in clinical and epidemiological studies [51] because it is applicable not only to evaluate self-rating health-related conditions or diseases among patients but also to initiate the health management of the general population [50] and detect the health problems related to work, housework, family, social life, and habits [52]. This instrument can be used to effectively and sensitively evaluate subjective health [53]; this instrument also successfully allows variations within and between illness groups [54]. In several domains, the NHP is also culture-free [55]; the NHP has also been used as a valid and reliable tool among the Chinese doctors [56].

The 38 items in the NHP were divided into six domains: physical mobility (8 items), pain (8 items), sleep (5 items), energy (3 items), social isolation (5 items), and emotional reactions (9 items). Each domain was independently scored from 0 to 100. Different weights were assigned to all items according to the paired comparison method. The respondents answered “yes” or “no” to each item on the basis of their actual situations; “yes” received corresponding weight scores, but “no” received no score. Higher scores indicated a poor health function in a particular domain. The QOL of respondents was calculated by generalizing the scores in the six domains.

### Analysis methods

The basic information of young clinical doctors was subjected to descriptive statistics or frequency analysis; the NHP results were also evaluated through reliability analysis. Cronbach’s alpha (α) indicated the internal consistency of the study. The scores in the 38 items were transformed and calculated into domain scores in accordance with the specific formulas. Comparisons were visually presented using bar or line charts. ANOVA and general linear regression model (GLM) methods were used to evaluate the effect of socio-economic variables, particularly age, gender, education, marital status, job title, monthly income, and work hours per day, on QOL and its six domains. Confirmatory factor analysis (CFA) was conducted to analyze the model fit of the NHP measurement.Ethics, consent and permissionsThe survey conducted with oral informed consent and the approval of the ethics committee of the University., in compliance with the principles of the Declaration of Helsinki. Interviewers informed each respondent of their right to refuse to participate, and of their right to refuse to answer any question, both initially and during the course of the research. After making sure that the respondents were clear about their rights and the possible consequences of this study, the interview would start. And authors would take the interpretation and responsibility for results involving human subjects in this study. Consent to publishBefore respondents filled in the questionnaire in this study, they had been told that their data would be used for academic research, and they ensured that their information filled in the questionnaire was in accordance with the actual situation. The interviewers are well trained to improve the quality of their data collection skills and meet the requirements of the medical ethics.  

## Results

### Sample characteristics

Table 1 shows the frequency analysis results of the basic information of the young clinical Chinese doctors. The mean age is 34.08 years; of the respondents, 58.4 % are men; furthermore, 10.1 % are in junior college or low levels. The three other categories are balanced at approximately 30 %. In addition, 70.4 % of the respondents are resident doctors (31.8 %) or physicians (38.6 %). The respondents holding the title of associate chief physician or chief physician are relatively few; these respondents account for 21.5 % and 8.1 % of the sample, respectively.

The distribution of monthly income is in an inverted U-shape; in this model, the minority of the respondents earn low or high salaries (<14 %). Approximately 76.1 % of the respondents receive 2,000–7,000 Yuan. According to the NBSC, the disposable incomes per month of the urban residents living in Shanghai, Jiangsu, and Zhejiang in 2013 are Chinese Yuan (CNY) 3,654.3, 2,711.5, and 3,154.2, respectively. In the current investigation, the monthly income of 36.9% respondents is lower than CNY 3,000. Therefore, the salary of the young clinical Chinese doctors is low.

On the basis of work hours per day, we found that only 8.7 % of the respondents, which correspond to the smallest percentage among the four categories, work less than 8 h a day, 42.4 % of the respondents work 12 h to 14 h a day, and 26.6 % of the respondents work for more than 15 h a day. The current legal prescription in China with regard to daily working time is 8 h. Moreover, 69 % of the doctors work more than 60 h each week, which is 50 % higher than the normal working time. Young clinical doctors work for extended hours.

### NHP reliability analysis

The Cronbach’s α coefficient of the total profile is 0.861 (> 0.8), which indicates that the NHP exhibits good internal reliability. Table 2 shows the coefficients of domains if these domains are deleted. All of the values are >0.500, which means that the internal consistency of the profile is acceptable. These values are less than the total Cronbach’s α coefficient (0.861); thus, the deletion of any domain is meaningless. Moreover, all of the correlation coefficients are significant at a 0.01 level. The values between 0.30 and 0.60 indicate that the domains yield low and moderate correlations.

**Table 2:** Cronbach’s α coefficients and Multi-dimensional Spearman correlation coefficients

Domains	Items	Cronbach’s α if domain is deleted	Physical mobility	Energy	Pain	Sleep	Social isolation	Emotional reactions
Physical mobility	8	0.648	1.000					
Energy	3	0.540	0.542^a^.	1.000				
Pain	8	0.610	0.476^a^.	0.441^a^.	1.000			
Sleep	5	0.564	0.447^a^.	0.352^a^.	0.393^a^.	1.000		
Social isolation	5	0.528	0.517^a^.	0.404^a^.	0.442^a^.	0.522^a^.	1.000	
Emotional reactions	9	0.670	0.590^a^.	0.548^a^.	0.571^a^.	0.495^a^.	0.539^a^.	1.000

^a^. Correlation is significant at the 0.01 level (2-tailed)

### NHP scores

Table 3 shows the descriptive statistics of the six NHP domains. The mean scores of all domains, except physical mobility and pain, are >50. Among the scores of these domains, the highest score is observed in sleep (57.01), followed by emotional reactions (54.47), social isolation (54.04), and energy (53.11). In the median values, all domains, except physical mobility and pain, are >50. The mode of physical mobility and pain is 0. However, the mode of the four other domains is 100, which shows that more respondents indicated severe function impairment in these four domains. Among these domains, physical mobility exhibits the largest absolute value of skewness. In terms of kurtosis, all domain values are <0. In addition, the minimum and maximum values of the six domains are the same.

**Table 3:** Descriptive statistics of NHP

	Physical mobility	Energy	Pain	Sleep	Social isolation	Emotional reactions
Mean	34.53	53.11	41.39	57.01	54.04	54.47
Median	31.75	60.80	38.48	60.53	57.34	50.06
Mode	0.00	100.00	0.00	100.00	100.00	100.00
Variance	656.64	1294.80	673.14	930.00	857.40	687.27
Skewness	0.52	−0.09	0.31	−0.20	−0.05	0.11
Kurtosis	−0.76	−1.29	−0.87	−1.04	−0.94	−1.10
Minimum	0.00	0.00	0.00	0.00	0.00	0.00
Maximum	100.00	100.00	100.00	100.00	100.00	100.00

### Effects of socio-economic variables on the six domains of QOL

#### Gender

**Fig. 1:**
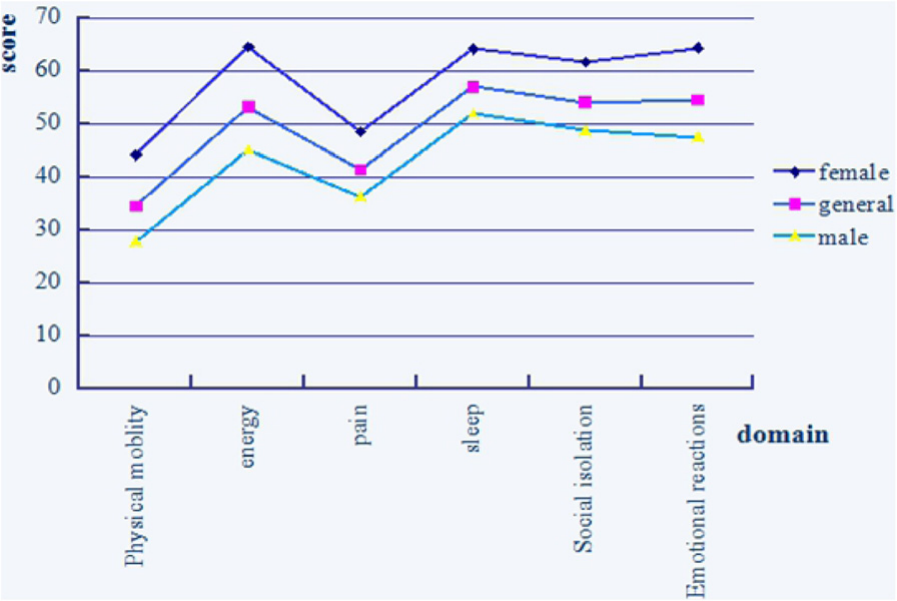
Mean score of young doctors under different genders

An independent sample *t* test was performed to analyze the effects of gender on the six QOL domains. The results indicate that gender significantly affects the QOL of young clinical Chinese doctors (*P* < 0.001). Figure 1 shows the comparison of the mean scores between females and males. The males yield lower scores than females, that is, the health functions of each domain of the males are better than those of the females. Among the scores of the domains, the scores of physical mobility and pain are the lowest; the scores of the four other domains are higher than those of the remaining domains (Fig. 1). In addition to the scores of physical mobility and pain, the scores of the females in the four other domains are >50.

#### Education

**Fig. 2:**
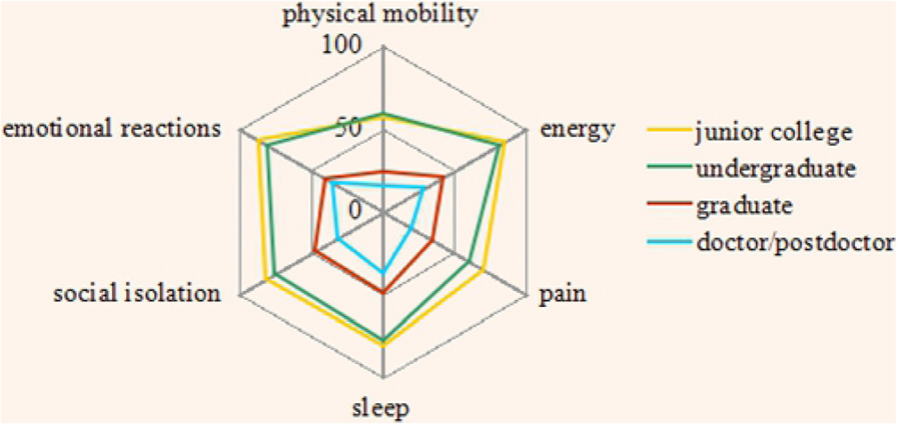
Domain scores of doctors with different educational levels

One-way ANOVA was performed to analyze the differences in the domain score of different educational categories. The results show that education significantly affects QOL at a 0.001 level. The comparison of the mean scores in the six domains across educational categories is shown in Fig. 2. The radar graph indicates the effect of education on QOL. The mean scores of the respondents at graduate and doctor/post-doctor levels are <50; this result indicates that their QOL is at the normal level. The scores of the doctors at the undergraduate level or those at junior level or less education degree are >50, particularly in the domains of energy and emotional reactions (>80). Therefore, the doctors with relatively low education experience poor QOL.

#### Job title

**Fig. 3:**
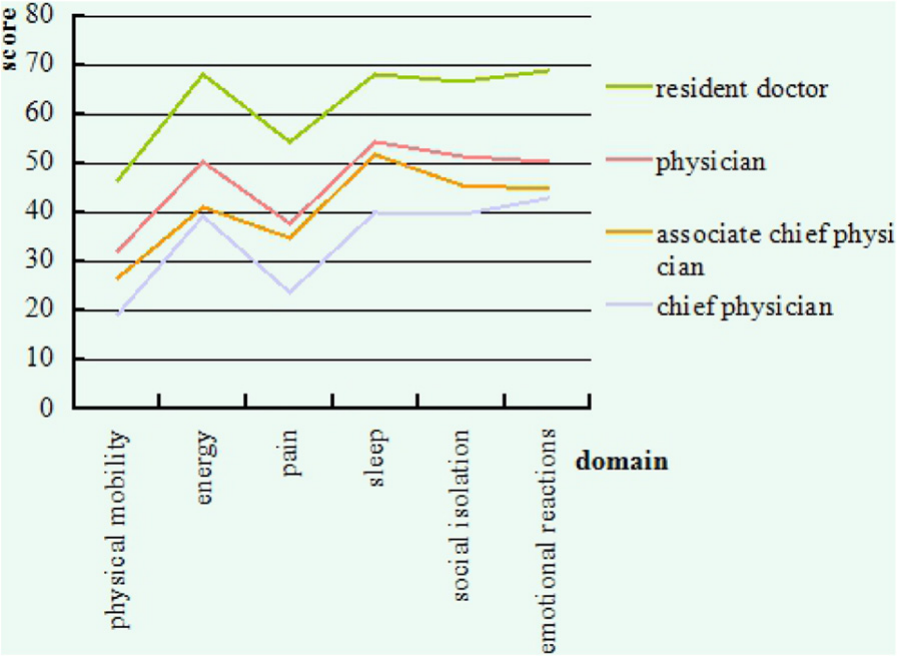
Comparisons of the domain score of the doctors with different job titles

ANOVA was performed to investigate the effect of job titles on QOL and to determine whether the mean differences between groups are significant. Figure 3 shows that the resident doctors yield the highest scores among all of the doctors who participated in this study. The scores decrease as the doctors reach a higher rank; this result indicates that the doctors with higher job titles experience better QOL. Among the scores of the domains, the highest scores of each line are detected in the sleep domain, followed by the energy domain; the lowest score of each line is found in the physical mobility domain.

#### Monthly income

**Fig. 4:**
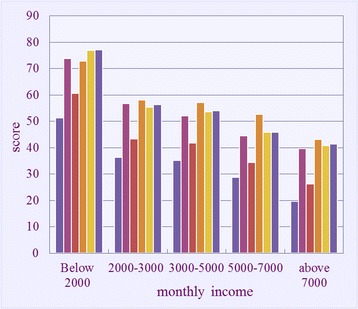
Effect of monthly income on the mean scores of the six domains

Figure 4 shows the mean score comparisons of the domain scores of the young clinical doctors with different wages 1. The domain scores of the doctors with a salary below 2,000 Yuan are higher than those with a salary above 2,000 Yuan. As the monthly income increases, the domain scores decrease; thus, the respondents with higher wages show better QOL. The doctors with salary below 2,000 Yuan yield scores of >50 in all of the NHP score domains; likewise, the doctors with a salary of 2,000–5,000 Yuan report scores of >50 in the four domains.

#### Work hours per day

Table 4 shows that the one-way *F*-values are relatively small. The effect of work hours per day on physical mobility, energy, and pain are significant at a 0.05 level; by contrast, the domains of sleep and emotional reactions are significant at a 0.01 level. Among the domains, social isolation does not show a significant mean difference. An increase in work hours likely exhibit a consistent increasing pattern of the domain scores, except the 9–11-h category in energy and emotional reaction domains.

**Table 4:** Mean values of six domains under different work hours per day

	N	physical mobility	energy	pain	sleep	social isolation	emotional relations
≤8 h	66	30.4461	50.2545	37.2773	54.1085	53.0336	52.9248
9–11 h	170	30.6868	46.0141	38.2916	51.3919	50.9036	49.1213
12–14 h	323	35.9457	54.8508	41.2909	56.2177	52.9842	54.3506
≥15 h	203	36.8358	57.2059	45.4840	63.9128	58.6633	59.6411
One-way F-value		2.728	3.505	3.072	5.768	2.518	5.153
Sig.		0.043	0.015	0.027	0.001	0.057	0.002

#### GLM models

GLM was used to analyze the effect of the socio-economic variables on the six QOL domains. Table 5 presents the results; each domain with a corresponding model is labeled 1 to 6. All of the models are significant at *P* < 0.001. The R square determination coefficients of the six models are between 0.3 and 0.7; this result indicates that the independent variables in the regression models can explain the 30–70 % of the variation of the dependent variables. The standard errors (S.E.) of the unstandardized coefficients are between 0 and 10, which are relatively small. In addition, the constants of all of the models are significant at the 0.001 level. Only the coefficient of Model 3 of age passes the significance test (*B* = −0.506, *P* < 0.05). The coefficient is negative; thus, the feeling of pain decreases with age. The coefficients of females do not show statistical significance.

**Table 5:** GLM results of six domains of QOL

Variables	Model 1	Model 2	Model 3	Model 4	Model 5	Model 6
	(physical mobility)	(energy)	(pain)	(sleep)	(Social isolation)	(emotional reactions)
(Constant)	66.904***	96.014***	86.694***	74.610***	96.652***	89.911***
	(5.784)	(9.397)	(6.327)	(8.578)	(7.551)	(5.087)
Age	−.293	−.429	−.506*	.206	−.359	−.048
	(.207)	(.337)	(.227)	(.307)	(.271)	(.182)
Female	2.608	2.463	−.857	−.993	−1.304	1.210
	(1.346)	(2.187)	(1.472)	(1.996)	(1.757)	(1.184)
Undergraduate	2.709	−1.928	−7.222**	−3.961	−3.370	−4.911*
	(2.427)	(3.942)	(2.654)	(3.599)	(3.168)	(2.134)
Graduate	−31.479***	−40.092***	−32.006***	−31.468***	−32.383***	−43.368***
	(2.455)	(3.988)	(2.685)	(3.640)	(3.204)	(2.159)
Doctor/post doctor	−41.636***	−52.749***	−46.357***	−42.877***	−47.570***	−48.382***
	(2.662)	(4.324)	(2.911)	(3.947)	(3.474)	(2.341)
Physician	−1.111	1.630	−1.248	-.624	1.168	.108
	(1.915)	(3.111)	(2.095)	(2.840)	(2.500)	(1.684)
Associate chief physician	−2.579	−1.711	.822	.354	1.107	.504
	(2.683)	(4.359)	(2.935)	(3.979)	(3.502)	(2.360)
Chief physician	−4.640	2.576	−3.774	−6.469	2.551	4.678
	(3.640)	(5.912)	(3.981)	(5.397)	(4.751)	(3.201)
Married	6.730***	−.564	2.734	−.527	2.991	−.331
	(1.886)	(3.064)	(2.063)	(2.797)	(2.462)	(1.659)
Divorced or others	4.534	4.302	6.069*	−1.848	3.866	−1.954
	(2.403)	(3.904)	(2.628)	(3.564)	(3.137)	(2.113)
Monthly income	−.258	.230	−.656	−.738	−1.781	−1.859**
	(.816)	(1.326)	(.893)	(1.211)	(1.066)	(.718)
Work hours per day	−1.208	−.476	−.410	1.177	−.666	.375
	(.752)	(1.221)	(.822)	(1.115)	(.981)	(.661)
R^2^	.570	.424	.498	.332	.439	.682

Figures in the table refer to the unstandardized Coefficients B; Figures in brackets represent Std. Error. All models pass the significance tests at the 0.001 level. * indicates P<0.05; ** indicates P<0.01; *** indicates P<0.001.

The coefficients of the undergraduates in Models 3 and 6 of education are significant at 0.01 and 0.05 levels, respectively. The coefficients of doctors with graduate and doctor/post-doctor degrees in all of the models are significant at a 0.001 level. The significant coefficients are negative. This finding indicates that a high educational degree yields a lower score than junior college or below educational degree; therefore, a better health function is observed in this domain. In particular, the variables, namely, graduate and doctor or post-doctor, yield the largest absolute value (>30). This result suggests that these two educational categories more strongly affect the QOL than other independent variables. The remarkable and significant effect of a high educational degree on the QOL improvement is emphasized.

All of the coefficients of the work hours per day are not significant (*P* > 0.05). In terms of marital status, only the coefficient of the married doctors in Model 1 (*B* = 6.730, *P* < 0.001) and the coefficient of the divorced doctors or doctors with other status in Model 3 (B = 6.069, *P* < 0.05) are statistically significant. Both of these coefficients are >6. Compared with the unmarried doctors, the married doctors exhibit a worse health function in physical mobility; the divorced doctors display a stronger feeling of pain.

### CFA

On the basis of the models or theories described in previous studies, we established the CFA measurement models of NHP. The six QOL domains are latent variables; this finding indicates that these domains can be described using the 38 items of NHP (observed variables). The six latent variables can also correspond to the QOL concept.

Table 6 shows the regression weights resulting from the CFA model. The Critical Ratio (*C. R)* values are >2, and the *S. E.* values are between 0 and 1, which are relatively small. All of the estimates are statistically significant. The referred category is emotional reactions. The coefficients of sleep, social isolation, and energy are >1. This result indicates that QOL exhibits a greater influence on these three domains than on emotional reactions.

**Table 6:** Regression weights of CFA model

			Estimate	S.E.	C.R.	P
emotional reactions	<−−-	QOL	1			
sleep	<−−-	QOL	1.106	0.136	8.114	***
social isolation	<−−-	QOL	1.133	0.136	8.317	***
physical mobility	<−−-	QOL	0.541	0.106	5.122	***
energy	<−−-	QOL	1.035	0.131	7.911	***
pain	<−−-	QOL	0.936	0.123	7.596	***

*S* sleep, *SI* social isolation, *ER* emotional reactions, *PM* physical mobility, *P* pain, *EL* energy level. *** indicates *P*<0.001

Table 7 shows the model fitting results of the CFA of the NHP. GFI, AGFI, IFI, TLI, and CFI are larger than the adaptive value of 0.90; PNFI and PCFI are less than the adaptive value of 0.50; therefore, the comparative model fitting is relatively good. Moreover, (*χ*^2^) = 918.321, *χ*^2^/DF = 1.391 < 2, RMR = 0.008 < 0.05, and RMSEA = 0.023 < 0.05; thus, the general model fitting is good.

**Table 7:** Model fitting indices of CFA

Fitting indices	Adaptive value	Results	Fitting
χ^2^		918.321	Yes
χ^2^/df	<2	1.391	Yes
RMR	<0.08: good	0.008	Yes
RMSEA	<0.05: excellent	0.023	Yes
GFI	>0.90	0.940	Yes
AGFI	>0.90	0.932	Yes
IFI	>0.90	0.940	Yes
TLI	>0.90	0.936	Yes
CFI	>0.90	0.940	Yes
PNFI	>0.50	0.766	Yes
PCFI	>0.50	0.882	Yes

*c* ^*2*^ Chi-square; c^2^/df = Chi-square / degree of freedom, *RMR* root mean square residual, *RMSEA* root mean square error of approximation, *GFI* goodness-of-fit index, *AGFI* adjusted goodness-of-fit index, *IFI* incremental fit index, *TLI* Tacker-Lewis index, *CFI* comparative fit index, *PNFI* parsimony normed index, PCFI parsimony comparative fit index

## Discussion

The mental health problems of doctors have increased the concern of many scholars. The mental health of Chinese doctors is in a similar situation. In the current Chinese society, obstacles such as frequent violence against medical workers and tense doctor–patient relationships affect the health of Chinese doctors. This study attempted to explore the general health condition of young clinical doctors in public hospitals in the developed cities of China to comprehensively study the health functions of the doctors in all domains and related risk factors. The results indicate that the young clinical Chinese doctors have high education levels, low job titles, moderate wages, and long work hours. Despite these findings, the doctors report poor QOL, sleep quality, and mental function.

On the basis of the descriptive statistical analysis results, we found that the sample investigation conducted in the three cities in East China reveals that the young clinical Chinese doctors have high educational levels but low job titles, moderate salaries, and long work hours. Clinical medicine is an occupation that requires comprehensive professional knowledge and highly trained medical practitioners. In China, medical students usually must finish five or six years of undergraduate professional studies [57]. The academic stress and psychological health of these medical students should be considered [19]. Therefore, the occupational pressure of doctors has been initially observed in their experiences as medical students. Doctors must undergo many years of training because the professional occupation requires comprehensive knowledge and skills. As such, the qualifications of these doctors are relatively low. Moreover, the job promotion mechanism of Chinese doctors requires certain years of practical experiences and series of examinations. For this reason, the job titles of young doctors are usually low. Compared with the salaries of other occupations, such as lawyers, with strict professional requirements, the salary of Chinese doctors are at a moderate level, but their work time is much more than the standard of 8 h. This event is related to the current situation of the Chinese medical system.  China is in great need for medical care because this country has the largest population in the world. However, the financial investment and policy support of the government are very weak and insufficient [58]. According to the Organization for Economic Co-operation and Development Health Statistics Yearbook 2014 supplemented by the country data from the Global Health Workforce Statistics of WHO, the physician density per 1,000 people in China was 1.7 in 2013; the physician density per 1,000 people in the UK and in the US in the same year were 2.7 and 2.6, respectively [59]. The use of limited medical resources to respond to these increasing needs is accounted for the work overload of Chinese medical workers.

In our study, the young clinical doctors yield scores of >50 in four domains other than physical mobility and pain. The comparison of the mean scores among the six QOL domains reveals that the young clinical Chinese doctors in the three developed cities exhibit poor sleep quality and disadvantaged mental functions but good physical functions. The average age of the respondents is 34.1 years, which is the age at which the careers of people move upward. The bodies of young doctors are full of energy, and these young doctors can do different things in life and work. Therefore, doctors with good physical functions are normal and understandable. However, these doctors do not experience high-quality sleep and suffer from psychological distress. Although their body is at the most energetic stage, these doctors also struggle with pressure and burnout to a worse extent that normal people. The self-rated health results of these doctors show the features of an aging population, and these results should be considered. This finding is consistent with that found in other countries [2, 8]; therefore, these conditions are determined by occupational characteristics or nature of work. Working in a medical system intrinsically damages the welfare of individuals [60].

Our results also indicate that the risk factors of the poor QOL of doctors are as follows: being female, low education, job titles, salary, and long work hours. Previous studies showed that the health of female doctors is poorer than that of males. For example, female doctors suffer from more minor physical ailments [12] and higher suicidal rate than male doctors [61]. The disadvantaged condition of the health-related QOL of females is found among many population groups [62–64]; thus, the health of females should be considered and a support-network system should be developed [65, 66]. Moreover, doctors with higher educational degree and job titles experience better QOL because doctors possess more professional knowledge and clinical experiences than those with lower educational degree and job titles; the former are also more mature in dealing with issues and show positive attitudes toward life and work than the latter. A low salary corresponds to the decreased wage of individuals; this finding is consistent with that in previous studies [67]. With high wages, individuals’ material life can be guaranteed, and QOL can be improved. Long work hours and high job demands not only damage physical health but also impair the well-being [68]. Long work hours also elicit a short-term negative effect on the psychological feeling and job performance of doctors [69].

The GLM results highlight the great and significant effect of education on the improvement of QOL. Furthermore, the empirical findings of this study likely contradict the common perception that older people feel more pain than younger ones. However, the research objects of the study are young clinical doctors. The oldest respondent in the sample is 45 years old. These doctors are young and at a strong stage in their lives; therefore, these young doctors unlikely suffer from senile and chronic diseases. However, many doctors are unaware of occupational health [69]. Although these doctors are considered as experts in their respective medical fields, they do not pay sufficient attention to their health conditions. When these doctors become ill, they continue to work but rarely seek normal medical consultations [70]. This phenomenon should be monitored. Moreover, the correlations between marital status and QOL remain unclear. The GLM results confirm and emphasize the importance of high education in QOL. The positive effect of education on health is obtained through the relationship among work, economic situation, and social capital resources [71, 72].

This study shows that the young clinical Chinese doctors working in public hospitals experience poor QOL and exhibit unfavorable mental functions. This condition is supposedly associated with the market-oriented reform of the Chinese medical system that began in the 1980s [73]. Public hospitals provide high-profit motivations to hire more qualified professionals and use more advanced equipment and drugs; however, these factors partially contribute to high medical expenses. Despite these benefits, the medical insurance system remains incomplete [74]. A great gap also exists between limited medical resources and high demands [58]. Public hospitals are commonly criticized because of their expensive costs and poor service quality [29], as well as the scarcity and misdistribution of the qualified workforce [25, 75]. Among the complaints of patients are the long waiting time for diagnosis and treatment, difficulty in reserving a qualified expert, and perfunctory attitude of doctors [76]. The difficulty in obtaining medical resources exacerbates the psychological condition of doctors [63].

In this context, tensions arise in the Chinese doctor–patient relationship. The violent incidents frequently observed in the present Chinese society, such as attacks or insults on medical workers and damages to hospital equipment, have been considered as risk factors that affect the normal operation of medical systems and the stability and harmony of society [77]. Doctor–patient conflicts also increase. Although Chinese doctors feel relatively unsafe, the phenomenon does not raise sufficient concern from hospital managers [78]. These violent events negatively affect medical workers; for instance, these workers may develop guilt and self-doubt, and such outcomes can reduce the quality of services [79].

Considering the multiple complicated factors, such as occupation features, social reality, and personal characteristics, affecting the health condition of Chinese doctors, institutional and individual institutions must improve the QOL of doctors throughout the country. The government should also strengthen the financial investment and policy support for medical services [80]. Given that the contextual factors are the bases that determine the work load and occupational stress of Chinese doctors, the working and living conditions of young clinical doctors in the developed cities in China can only be completely improved when the issues on resource allocation, insurance reimbursement, and service quality in Chinese medical care systems are resolved. Considering the tense doctor–patient relationship and arising conflicts, we found that the safety of work environments and the occupational support for doctors face serious challenges. Thus, hospital health management should create a supportive work environment, improve the corresponding psychological capital [30], and promote doctor–patient communication. The expectations and requirements of society, patients, and families should be practical. Young clinical doctors can focus on skills and service improvement when they work in less strict social environments where these doctors do not need to bear considerable external pressure. As individuals, doctors should learn further insights into occupational health and acquire skills to relieve pressure. In appropriate circumstances, these doctors may consult social workers [81].

This study has some limitations. First, in the sample investigation, public hospitals are selected from the three relatively developed cities in East China. Therefore, our findings may not be applicable to doctors working in less developed areas or in private hospitals. Second, the study only analyzed the cross-sectional data and did not explore the dynamic nature of the QOL of doctors. At any specific or different time, such as when vicious violence against doctors unexpectedly occurs, the QOL of young clinical Chinese doctors may greatly change. Third, only the effects of several socio-economic variables on QOL were discussed. Against the complicated background of the current Chinese medical system, external factors, such as doctor–patient relationship and social support, may elicit more immediate effects on the health of doctors. Notably, this study did not include the variables related to the medical field, and these variables can be associated with the work-related stress of doctors. Thus, this area can provide new study perspectives. Fourth, the education of the samples in this study is conditioned because of the age limit of these samples. In addition, the research objects are young clinical doctors aged 15–45 years, and these doctors constitute the professional group with the specific physiological structures or psychological characteristics of this age group. Therefore, our results may not be expanded to older populations.

## Conclusion

On the basis of the medical system reform and doctor–patient tension in the current Chinese society, we used the reliable and valid NHP to measure the QOL of young clinical Chinese doctors in public hospitals. This study deviated from the single perspectives on mental problems in previous studies and focused on the general health condition in terms of QOL. Our findings were explained in the context of the current Chinese medical environment. The results indicate that the young clinical Chinese doctors have high education levels, low job titles, moderate wages, and long work hours. These doctors report poor QOL, sleep quality, and mental functions. Furthermore, being female, low education, job titles, salary, and long work time were identified as the risk factors of the QOL of doctors. This finding may be related to multiple factors, such as occupation features, social reality, and personal character, including the controversial medical system and the tense relationship between doctors and patients. The efforts and dedication of all institutions and individuals in the whole country are necessary to improve the QOL of young clinical Chinese doctors. The fundamental issue involves the mechanism by which the current medical care system in China can be reformed; in addition to these mechanisms, the methods by which the work environment of young clinical doctors can be improved and their occupational stress can be relieved should be investigated.

## Footnotes

1 Job titles in Chinese public hospitals from a low rank to a high rank are resident doctor, physician, associate chief physician, and chief physician. Doctors must pass an exam and accumulate certain years of working experience to be promoted. The assessment indices mainly refer to the clinical and academic performances of these doctors.
